# Human blood RNA stabilization in samples collected and transported for a large biobank

**DOI:** 10.1186/1756-0500-5-510

**Published:** 2012-09-18

**Authors:** Nur Duale, Gunnar Brunborg, Kjersti S Rønningen, Thomas Briese, Jeanette Aarem, Kaja K Aas, Per Magnus, Camilla Stoltenberg, Ezra Susser, W Ian Lipkin

**Affiliations:** 1 The Norwegian Institute of Public Health, Oslo, Norway; 2 The Mailman School of Public Health, Columbia University, New York, NY, USA; 3 New York State Psychiatric Institute, New York, NY, USA; 4 Present address: Oslo University Hospital, Rikshospitalet, Oslo, Norway

**Keywords:** Tempus tubes, PAXgene tubes, Cord blood, Quality control, RNA stabilization, Biobank, Blood RNA, Gene expression

## Abstract

**Background:**

The Norwegian Mother and Child Cohort Study (MoBa) is a nation-wide population-based pregnancy cohort initiated in 1999, comprising more than 108.000 pregnancies recruited between 1999 and 2008. In this study we evaluated the feasibility of integrating RNA analyses into existing MoBa protocols. We compared two different blood RNA collection tube systems – the PAXgene™ Blood RNA system and the Tempus™ Blood RNA system - and assessed the effects of suboptimal blood volumes in collection tubes and of transportation of blood samples by standard mail. Endpoints to characterize the samples were RNA quality and yield, and the RNA transcript stability of selected genes.

**Findings:**

High-quality RNA could be extracted from blood samples stabilized with both PAXgene and Tempus tubes. The RNA yields obtained from the blood samples collected in Tempus tubes were consistently higher than from PAXgene tubes. Higher RNA yields were obtained from cord blood (3 – 4 times) compared to adult blood with both types of tubes. Transportation of samples by standard mail had moderate effects on RNA quality and RNA transcript stability; the overall RNA quality of the transported samples was high. Some unexplained changes in gene expression were noted, which seemed to correlate with suboptimal blood volumes collected in the tubes. Temperature variations during transportation may also be of some importance.

**Conclusions:**

Our results strongly suggest that special collection tubes are necessary for RNA stabilization and they should be used for establishing new biobanks. We also show that the 50,000 samples collected in the MoBa biobank provide RNA of high quality and in sufficient amounts to allow gene expression analyses for studying the association of disease with altered patterns of gene expression.

## Findings

### Background

The Norwegian Mother and Child Cohort Study (MoBa) is a nation-wide population-based pregnancy cohort initiated in 1999 [1,2]. The MoBa holds more than 108,000 pregnancies recruited between 1999 and 2008; the last child to be included was born in June 2009. In addition to detailed questionnaire data, biological materials from the mother, the father and the child (umbilical cord blood) in the form of whole blood and plasma, have been collected and are stored in a biobank at the Norwegian Institute for Public Health (NIPH) together with extracted DNA, for future use [2]. The DNA is routinely extracted from the blood samples from each participant and frozen in aliquots. In 2005 funds were obtained from the US National Institutes of Health through the Autism Birth Cohort to initiate RNA collection for the purpose of gene expression analyses. The challenge in implementing RNA collection was to devise a new protocol that would be feasible in maternity units and during transportation - and at the same time the existing routines and logistics of the cohort should be adhered to as closely as possible. There are several reports on the *ex vivo* instability of RNA transcripts [3-5], and it is therefore very important to stabilize blood RNA during sample collection, transport, and storage [6], in order to obtain reproducible gene expression results.

The overall goal of this study was to establish a practical protocol for sampling, handling, transportation and storage of adult and cord blood RNA. On this background, we systematically compared the RNA quality, quantity, and the RNA transcript stability of two commercially available RNA stabilizing technologies PAXgene™ Blood RNA system (PreAnalytiX, QIAGEN/BD, Hombrechtikon, Switzerland), and Tempus™ Blood RNA system (Applied Biosystems, Foster City, CA). We then investigated the effects of transportation and blood volumes on the RNA transcript stability of six selected genes (*CDKN1A, FOS, IL8, MYC, IL1B,* and *TP53*) using quantitative real-time PCR (RT-qPCR). These genes were selected based on literature search [7,8].

## Results and discussion

In MoBa, the combination of biological specimens and questionnaire data on lifestyle and exposures provide unique possibilities to study the effects of many factors of relevance for pregnancy outcomes and health [1]. Blood-based large biobanks such as MoBa, and multi-center studies, increasingly incorporate gene expression profiling studies in order to get insight into the biological mechanisms triggered by gene-environment interactions and disease. However, collection, transportation, processing and storage of blood samples may represent logistical and technical challenges [9,10]. Therefore, establishment of robust and standardized protocols is a prerequisite in order to reduce the impact which preanalytical sample handling may exert on RNA quality and stability. This is particularly important when collecting blood samples for large-scale biobanks, where the associated costs are very high.

In this study, we investigated important input factors for large-scale collection of blood from adults and children (umbilical cord blood). Although high-quality RNA can be prepared using fresh blood that is processed immediately, this option is hardly realistic when sampling for a large-scale biobank.

### Comparison of two RNA stabilizing systems, PAXgene and Tempus

**Figure 1:**
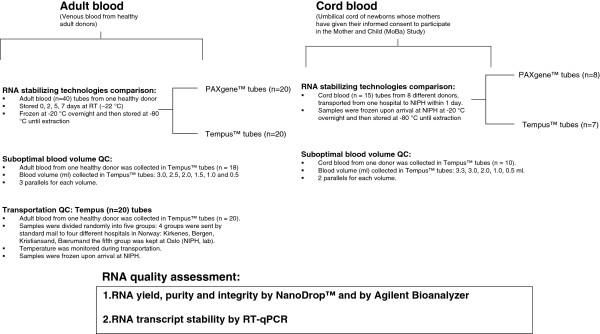
Flow chart: experimental design.

**Figure 2:**
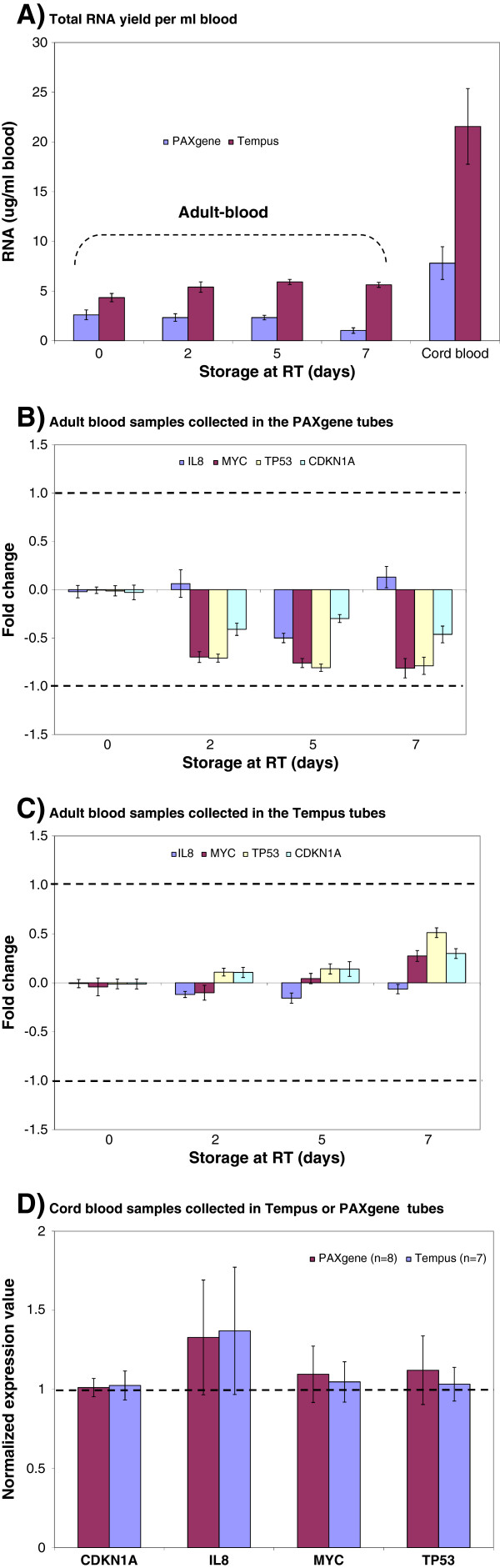
**Comparison of PAXgene and Tempus tubes.** Blood (adult or cord blood) was collected either in the PAXgene tubes or in the Tempus tubes, and stored at room temperature for the indicated number of days, followed by total RNA isolation. Five tubes were used at each time point (n=5). **A**) The RNA yield from the cord blood and the adult blood samples collected either in the PAXgene tubes or in the Tempus tubes. Each bar represents the average RNA yield per tube ± SE. **B**) Relative transcript levels of four genes from adult blood samples collected in the PAXgene tubes and stored for up to seven days at room temperature; **C**) Relative transcript levels of four genes from adult blood samples collected in the Tempus tubes and stored for up to seven days at room temperature. The 0 day samples were used as reference samples (calibrators); hence all other samples were compared against the reference samples. Each bar represents the average log2-transformed fold change values and the error bar indicates ± SE. The dashed lines indicate ± 2 – fold change. The fold change detected for the genes were within ± 2 – fold. **D**) The normalized expression values of four genes from cord blood samples collected in the PAXgene tubes (n=8) and in the Tempus tubes (n=7). Each bar represents the normalized expression values [2^-Δ*Cq* (sample)^; where the Δ*Cq* (sample) ± *Cq* (gene) – *Cq* (internal control)] and the error bar indicates ± SE.

Two commercially available blood RNA stabilizing systems, the PAXgene blood RNA system and Tempus blood RNA system, were available as evacuated tubes containing RNA stabilizing reagents, and both PAXgene [7,11,12] and Tempus [13] blood RNA systems have been shown to provide efficient stabilization of the blood RNA for several days at room temperature. In this study, the two systems were systematically evaluated regarding their performance to stabilize blood RNA; i.e. preserving the RNA quality, yield, and the RNA transcript stability of four selected genes (*CDKN1A*, *IL8*, *MYC*, and *TP53*). The study design for blood collection is outlined in Figure 1. The RNA qualities and yields for samples collected directly into PAXgene or Tempus tubes are shown in Figure 2A and Table 1. The average total RNA yields for adult and cord blood samples collected in the PAXgene tubes were 2.1 ± 0.2 μg and 7.8 ± 1.7 μg per ml blood, respectively, while the average total RNA yields for samples collected in the Tempus tubes were 5.3 ± 0.2 μg per ml blood for adult blood and 21.6 ± 3.8 μg per ml blood for cord blood (Figure 2A). The difference in yield per ml blood between the two types of collection tubes was significant (p < 0.05). The obtained RNA yields for blood samples collected in the Tempus tubes were consistently higher than for samples collected in the PAXgene tubes (Figure 2A). Higher RNA yields from the Tempus tubes compared with PAXgene tubes have been previously reported [14,15]. The RNA yield obtained from cord blood samples was 3–4 times higher than from adult blood using both types of tubes (Figure 2A).

**Table 1:** Comparison of blood samples collected in PAXgene and in Tempus tubes

**A) Adult blood**				
**Storage at RT (days)**	**RIN value**	**OD 260/280 ratio**	**OD 260/230 ratio**	**Number of tubes**
PAXgenes tube				
0	8.0 ± 0.3	2.12 ± 0.02	1.56 ± 0.31	5
2	8.0 ± 0.3	2.10 ± 0.03	1.56 ± 0.62	5
5	8.2 ± 0.3	2.12 ± 0.02	1.37 ± 0.73	5
7	8.2 ± 0.3	2.09 ± 0.03	1.42 ± 0.70	5
Average	8.10 ± 0.3	2.11 ± 0.02	1.48 ± 0.61*	20
Tempus tubes				
0	8.0 ± 0.3	2.12 ± 0.07	2.01 ± 0.14	5
2	8.2 ± 0.3	2.12 ± 0.07	2.01 ± 0.14	5
5	8.0 ± 0.3	2.14 ± 0.08	2.09 ± 0.08	5
7	8.2 ± 0.3	2.11 ± 0.04	2.08 ± 0.06	5
Average	8.10 ± 0.3	2.12 ± 0.07	2.05 ± 0.11	20
**B) Cord blood**				
	**PAXgene (n = 8)**		**Tempus (n = 7)**	
RIN value	8.1 ± 0.2		8.0 ± 0.2	
OD 260/280 ratio	2.1 ± 0.1		2.0 ± 0.3	
OD 260/230 ratio	1.6 ± 0.5*		2.0 ± 0.8	

Notes.* RNA from PAXgene tube-collected samples have significantly lower OD 260/230 ratio compared to RNA from Tempus tube-collected samples, p < 0.05. The cord blood samples were transported to NIPH (lab) within one day. n = number of tubes.

The integrity and purity of RNA can be used to evaluate the quality of sample handling and processing. A RIN-value higher than five is considered as indicating good quality RNA whereas RIN-values above eight are considered ideal for downstream applications [4,5]. When we investigated the RNA quality, we observed similar and comparable average OD 260/280 ratios and average RIN values for all adult and cord blood samples collected in Tempus and PAXgene tubes (p > 0.05), but the average OD 260/230 ratio for the samples collected in the PAXgene tubes was lower (p < 0.05) than for samples collected in Tempus tubes (Table 1). It has previously been reported that OD 260/230 ratios are higher for Tempus tubes compared with PAXgene tubes [15]. The observed differences in the average OD 260/230 ratio between the two systems may be due to the PAXgene elution buffer which may contain high salt contents. Storage of PAXgene and Tempus samples at room temperature for up to seven days had little effect on RNA quality, although a slight decrease in RNA yield (p < 0.05) was observed for adult samples collected in the PAXgene tubes at seven days of storage (Figure 2A). The RNAs isolated from both PAXgene and Tempus had average RIN-values of approximately eight, and average OD 260/A280 ratios were above 1.8 (Table 1). Based on these data, high-quality RNA could be extracted from blood samples stabilized with both PAXgene and the Tempus system.

The RNA transcript stability and potential alteration of the transcript level for four genes (*CDKN1A, IL8, MYC,* and *TP53*) were investigated by RT-qPCR (Figure 2B-D). Storage of both PAXgene and Tempus tubes at room temperature for up to seven days had some effect on RNA transcript stability; there were clear storage related changes in the transcript levels. For adult blood samples collected both in PAXgene and Tempus tubes, storage for up to seven days at room temperature had effects on the relative transcript levels for the four genes when compared to the day 0 samples (Figure 2B and C). Storage effects were more pronounced in samples collected in the PAXgene compared to the Tempus tubes. However, the observed transcript level changes were within ±2–fold for both types of tubes (Figure 2B and C). The non-normalized raw *Cq*-values for the four genes also illustrate similar trends (Additional file 1A and B). The differences in the *Cq*-values between stored and day 0 samples for each gene were very small. For cord blood samples, the average transcript levels for the four genes were comparable between the Tempus and the PAXgene tube samples, and the difference between the two systems (Figure 2D and Additional file 1C) was not significant (p > 0.05). Storage for up to 7 days exceeds the maximum period (3–5 days) as recommended by both manufacturers. The recommended storage time may be too strict, and a more flexible protocol – also giving RNA of acceptable quality - can more conveniently be implemented in multi-center studies.

Given the results of this initial investigation where samples collected in the Tempus tubes gave higher RNA yields with comparable quality when compared to PAXgene tubes, we selected the Tempus Blood RNA system for MoBa and began collecting cord blood in Tempus tubes in June 2005. Since then, approximately 50,000 samples have been collected and stored at −80°C.

### Transportation QC: The effect of transportation on RNA quality and transcript stability

**Figure 3:**
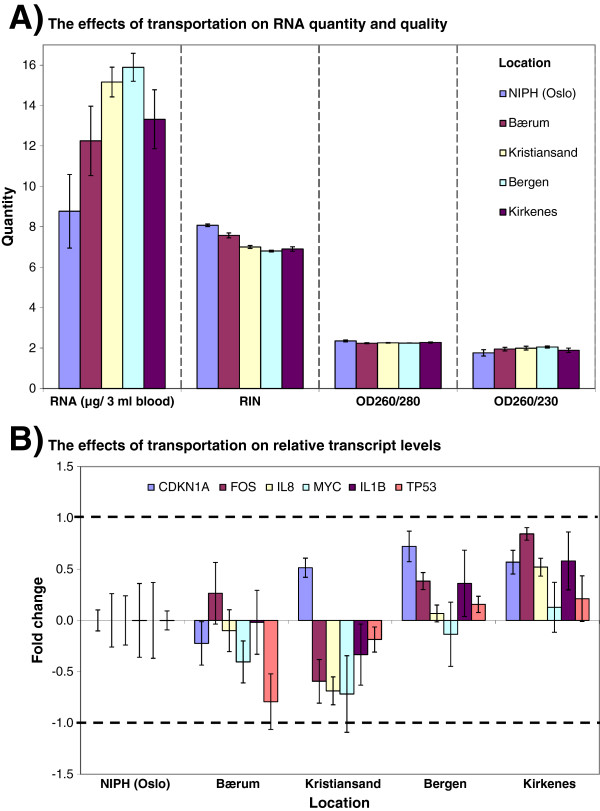
**The effects of transportation on RNA, quantity, quality and transcript stability.** Adult blood from one healthy donor was collected in Tempus™ tubes (n = 20). Samples were divided randomly into five groups (n = 4 tubes per group): four groups were sent by standard mail to four different hospitals in Norway, and the fifth group was kept at NIPH (Oslo/lab). Temperature was monitored during transportation. **A)** The effects of transportation on RNA quality and yield; **B)** The effects of transportation on RNA transcript levels of six genes. Samples kept at NIPH were used as reference samples; hence all other samples were compared against the reference samples. Each bar represents the average log2-transformed fold change values and the error bar indicates ± SE. The dashed lines indicate ± 2 – fold change.

The maternity units where sample collection took place are located all over Norway, and from a few hours up to several days may be required before the blood samples arrive for processing at the NIPH biobank. The distance between blood collection location and the biobank, and the associated differences in duration and temperature fluctuations during transportation, may affect the quality of the obtained RNA and subsequently the gene expression data. We investigated the effect of transportation on RNA quality and transcript stability of six genes (*CDKN1A, FOS, IL8, MYC, IL1B,* and *TP53*) which have been used in earlier studies [7,8]. Whole venous blood from one healthy donor (Figure 1) was collected by venous puncture into 20 Tempus tubes. The tubes were divided randomly into five groups, of which four were sent by standard mail to four maternity units in Norway whereas the fifth was kept at room temperature (24.4 ± 1.9°C) in the NIPH laboratory in Oslo. Samples were returned from the maternity units to NIPH and kept there at room temperature until all four sets had arrived; they were then frozen at −20°C at the same time followed by transfer to −80°C 24 hours later. The temperature logs showed values during transportation ranging from 3.4°C to 27.1°C; none of the samples were - or had been - frozen (Additional file 2A). Samples sent to Kristiansand had the highest mean temperature (20.28 ± 5.58°C), while those to Bærum had the lowest mean temperature (16.96 ± 8.55°C). The mean temperature differences between the transported samples and samples kept at NIPH are presented in Additional file 2A. Samples sent to Bærum had the highest mean temperature difference (− 8.0°C) relative to the samples kept at NIPH. RNA was extracted from all samples and analyzed for quality, and RT-qPCR was carried out to measure the relative transcript levels for the six selected genes. Figure 3A shows that transportation of samples by standard mail apparently affected RNA quality, especially the RNA integrity expressed as RIN value. There was a statistically significant location-dependent reduction in the average RIN value (p < 0.05); samples kept at NIPH had the highest RIN values; the lowest were from the Kirkenes and Bergen samples. The temperature recordings were divided into 12-hour intervals and averaged (Additional file 2A). Compared to the other samples, those from Bærum had much lower temperature (≤ 6°C) for two 12-hour time intervals. This might be relevant for the observed RNA quality of these particular samples (Figure 3A and Additional file 2B). In any case, the average RIN value for each group was greater than six, which is considered acceptable for many RNA assays.

Figure 3B shows the relative transcript levels for the six genes. Transportation of the samples apparently affected the relative transcription levels of the genes. Samples sent to Kirkenes or Kristiansand showed the highest changes compared to the samples kept at NIPH (control samples) (Figure 3B). However, the observed transcript level changes were again within ± 2–fold. The non-normalized raw *Cq*-values for the six genes and the average raw *Cq*-values ± SD for each gene are presented in Additional file 2D. Differences in the *Cq*-values between transported samples and samples kept at NIPH for each gene were small. A significant finding is that higher RNA yields were obtained from transported samples compared to samples kept at NIPH (Figure 3A and Additional file 2C); the reason for this is unclear. Samples can be shaken during transportation due to mechanical movements and vibrations, and temperature fluctuations occur (Additional file 2C), which may affect the RNA extraction efficiency.

Overall, transportation of the samples by standard mail had some effects on RNA yield, quality and RNA transcript stability. Temperature fluctuations, mechanical movements or vibrations during the transportation of samples may have contributed to the differences (Figure 3 and Additional file 2). However, the observed differences between transported samples and samples kept at NIPH were minor, and the obtained RNA quality from transported samples was considered acceptable for many RNA assays. Our results therefore suggest that blood samples collected in Tempus tubes can be sent by standard mail, under the conditions employed in this study.

### Suboptimal blood volume QC: The effect of suboptimal blood volume on RNA quality and transcript stability

**Figure 4:**
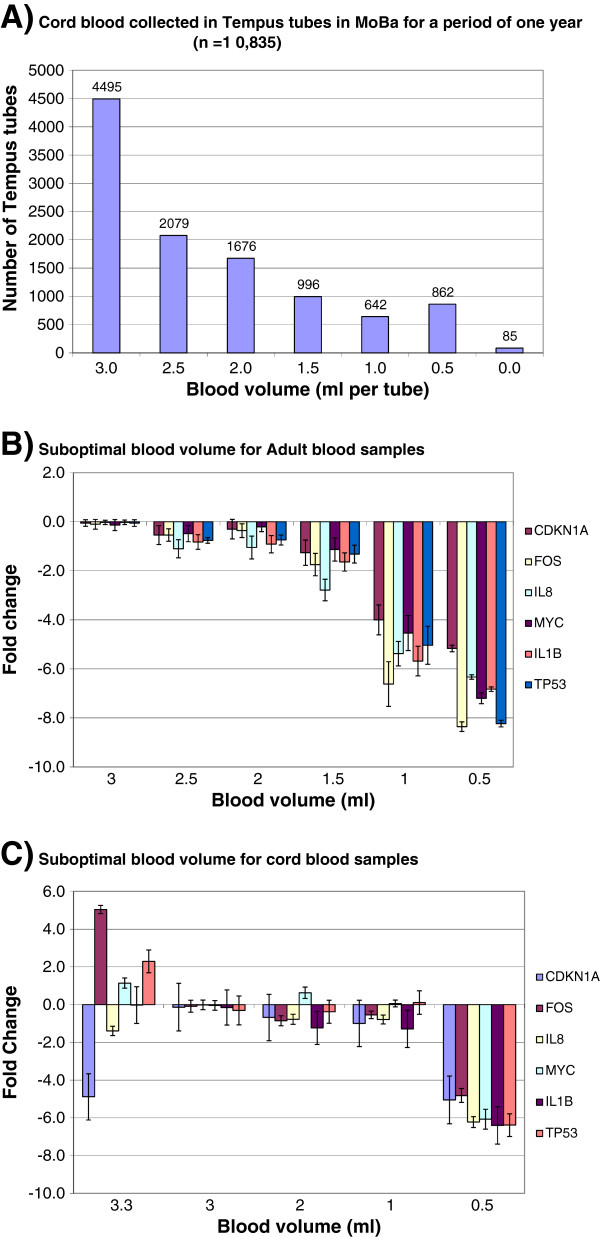
**The effect of suboptimal blood volume collected in the Tempus tubes on RNA quality and transcript stability. A**) Cord blood collected in Tempus tubes from maternity units in a period of one year (n = 10 853). **B**) The effects of suboptimal volume of adult blood collected in the Tempus tubes on relative transcript level for six genes. Adult blood from one healthy donor was collected in Tempus tubes (n = 18). The following blood volumes (3.0, 2.5, 2.0, 1.5, 1.0 and 0.5 ml) were collected in the Tempus tubes (n = 3; three parallels tubes for each blood volume). **C**) The effects of suboptimal volume of cord blood collected in the Tempus tubes on relative transcript level for six genes. Cord blood from one donor was collected in Tempus tubes (n = 10). The following blood volumes (3.3, 3.0, 2.0, 1.0, and 0.5 ml) were collected in the Tempus tubes (n = 2; two parallels tubes for each blood volume). Samples with 3 ml blood per tube were used as reference samples; hence all other samples were compared against the reference samples. Each bar represents the average log2-transformed fold change values and the error bar indicates = SE. Since some of the samples had low RNA quality, selecting stably expressed reference genes for normalization was a challenge. We therefore used the average *Cq* value for each gene to normalize the samples: Δ*Cq* (sample) = *Cq* (gene) – *Cq* (average); these values where converted into linear scale, 2^-ΔCq^. Fold changes were calculated by dividing Δ*Cq* value of the sample by the Δ*Cq* value of the reference sample.

Regular sample collections for the MoBa biobank were carried out in busy maternity units. A considerable portion of the samples collected contains blood volumes less than the optimal volume recommended by the manufacturer (Figure 4A); this is a common problem when collecting cord blood for research purposes. Figure 4A shows cord blood collected in Tempus tubes from different maternity units during a period of one year (n = 10835). We observed that ~ 20% of the tubes contained less than 2 ml blood. We investigated whether the recommended blood–to–RNA stabilizing reagent ratio was critical for blood RNA quality and RNA transcript stability, and observed a significant (p < 0.05) blood volume-dependent reduction in RNA yield, RIN value, and OD 260/280 and OD 260/230 ratios (Table 2A). The RNA blood chemistry seems to collapse when the blood volume in the tubes was below 2 ml. This was true for adult blood, but suboptimal blood volumes were not critical when collecting cord blood (Table 2B).

**Table 2:** The effect of blood volume in the Tempus tubes on RNA quality

**Blood volume (ml)**	**Yield total RNA (ug)**	**RNA Integrity Number (RIN)**	**OD260/280 ratio**	**OD2602/230 ratio**
A) Adult blood samples (n = 3)*				
3.0	7.90 ± 1.50	8.20 ± 0.06	2.19 ± 0.02	2.10 ± 0.23
2.5	3.38 ± 0.90	8.13 ± 0.27	2.24 ± 0.05	0.91 ± 0.46
2.0	2.94 ± 0.91	8.40 ± 0.08	2.12 ± 0.08	1.05 ± 0.53
1.5	1.38 ± 0.59	3.53 ± 2.53	2.51 ± 0.08	0.64 ± 0.32
1.0	0.55 ± 0.50	1.13 ± 0.13	1.19 ± 0.44	0.38 ± 0.38
0.5	0.07 ± 0.03	1.00 ± 0.00	0.77 ± 0.39	0.05 ± 0.05
B) Cord blood samples (n = 2)				
3.3	79.65 ± 4.76	7.15 ± 0.78	2.15 ± 0.00	2.12 ± 0.00
3.0	74.60 ± 1.23	7.50 ± 0.65	2.17 ± 0.00	2.15 ± 0.01
2.0	44.42 ± 1.91	9.10 ± 0.00	2.18 ± 0.01	2.09 ± 0.01
1.0	33.92 ± 3.13	9.45 ± 0.04	1.98 ± 0.01	1.61 ± 0.02
0.5	4.29 ± 0.11	1.03 ± 0.03	1.25 ± 0.03	0.53 ± 0.01

*Notes.* *There are significant blood volume dependent reductions in the RNA yields and in the RNA qualities (RIN, OD 260/280 and OD 260/230 ratio), p < 0.05. n = number of tubes.

The relative transcript levels for the six genes are shown in Figure 4B and C. We observed blood–volume dependent reduction in the relative transcript levels for the six genes compared with samples with 3 ml blood volume (Figure 4B and C). Samples with volumes less than 1.5 ml for adult blood and less than 1.0 ml for cord blood showed the highest changes in the relative transcript levels for the six genes, compared with the optimal 3 ml samples (Figure 4B and C). The non-normalized raw *Cq*-values for adult and cord blood samples show a similar trend (Additional file 3A and B). Furthermore, overfilling the Tempus tubes with cord-blood, i.e. more than 3 ml blood per tube, seems to affect the apparent RNA transcript stability, suggesting that the blood–to–RNA stabilizing reagent ratio limit was exceeded (Figure 4C). Our results indicate that the blood–to–RNA stabilizing reagent ratio is very important for blood RNA quality and transcript stability for adult blood samples, whereas the blood volume is less critical for cord blood samples. Hence, collection of 3 ml of blood per Tempus tube will always be the highly recommended optimal blood volume, but this volume is not always possible when blood collection is carried out in a busy maternity unit. For adult blood samples it is advisable to collect 3 ml blood per Tempus, whereas for cord blood samples, minor deviations from the optimal volume per tube may be acceptable.

## Conclusions

Our results indicate that blood samples in a large biobank such as MoBa can be used to obtain high-quality RNA suitable for gene expression analysis, provided that special collection tubes are used. The quality of the RNA isolated from such tubes is crucial for successful transcript profiling analyses. Transportation of samples by standard mail apparently affected the RNA quality and RNA transcript stability, but the changes were moderate and the overall RNA quality of the transported samples was good. Some unexplained changes in gene expression changes seem to be associated with suboptimal blood volumes collected in the tubes. Overall, we demonstrate that satisfactory amounts of high-quality RNA can be achieved from samples routinely collected in a large biobank. This is good news for future projects studying association of disease with altered patterns of gene expression.

## Methods

### Blood collection and study design

Whole blood was collected at a maternity unit in Oslo from the umbilical cord of newborns whose mothers had given their informed consent to participate in the MoBa project. Venous blood samples were obtained via phlebotomy from healthy adult donors among the NIPH staff after informed consent. The study design for blood collection is outlined in Figure 1. Samples were collected either into Tempus™ Blood RNA Tubes (space for 3 ml blood) (Applied Biosystems, Foster City, CA) or PAXgene Blood RNA Tubes (space for 2.5 ml blood) (PreAnalytiX, Qiagen, Germany). Samples collected at NIPH (Oslo) were stored for 2 h at room temperature, then at – 20°C for 24 h and finally transferred to – 80°C for long-term storage. Samples shipped by standard mail were then stored at – 20°C for 24 h after arrival at NIPH, and then transferred to – 80°C for long term storage. All samples were handled as recommended by the manufacturers prior to processing.

The adult blood samples collected to study the effects of transportation were shipped to 4 different locations in Norway, with instructions to treat the tubes the same way (temperature, storage) as tubes collected in the local maternity unit, and then to return the tubes to NIPH as it would be normally done with the collected tubes. The four sets were shipped by standard mail to the maternity units in Bærum (~15 km from Oslo), Kristiansand (~ 320 km), Bergen (~ 520 km) and Kirkenes (~ 2000 km). A fifth set of samples was let at room temperature at NIPH (Oslo/lab). The tubes were sent to the hospitals and then returned by the local staff to NIPH, to comprise a worst-case scenario of double the ordinary mail transportation time. Upon arrival at NIPH samples were kept at RT until the last sample arrived and then they were all frozen at the same time. Temperature was monitored during the transportation using small sensors with memory (Tinytag Talk2, Precision Technic Nordic, Rødovre, Denmark). The Regional Ethics Committee and the Data Inspectorate approved the MoBa study, and participants gave their informed consent.

### RNA extraction

Total RNA from whole blood collected in PAXgene tubes was extracted according to the manufacturer’s instructions and included DNase I treatment (PreAnalytiX, QIAGEN/BD, Hombrechtikon, Switzerland). Total RNA from blood collected in Tempus tubes was extracted using the Tempus™ 6-Port RNA Isolation Kit Protocol on an ABI PRISM® TM 6100 Nucleic Acid PrepStation according to the manufacturer’s instructions and included DNase I treatment and addition of 1X PBS bringing the total volume to 12 ml prior to processing (Applied Biosystems, Foster City, CA).

An aliquot of 5 μl of each extracted total RNA was used for RNA quality control assessments, while the remaining RNA sample was stored at – 80°C until use.

### RNA QC

The concentration of extracted total RNA was measured using NanoDrop ND-1000 spectrophotometer (Fisher Scientific, Norway). RNA purity was estimated by examining the OD 260/280 and the OD 260/230 ratios. The RNA integrity was assessed by Agilent 2100 Bioanalyzer using the Eukaryote total RNA 6000 Nano LabChip kit and Eukaryote total RNA Nano assay according to the manufacturer’s instructions (Agilent Technologies, Palo Alto, CA). The RNA integrity numbers (RIN) were calculated using the Agilent 2100 Expert Software (RIN = 1; low RNA quality to RIN = 10; highest RNA quality).

### Quantitative real-time PCR measurement

Total RNA (500 ng) from samples was used as template for the synthesis of cDNA using the High Capacity cDNA Reverse Transcription Kit (Applied Biosystems, Foster City, CA) according to manufacturer’s protocol. The amplification reactions were carried out in an Eppendorf MasterCycler (Eppendorf, Hamburg, Germany) with the following steps: 10 minutes at 25°C, 2 hours at 37°C and finally, 5 minutes at 85°C. The quality of the cDNA was assessed by the NanoDrop ND-1000 spectrophotometer (Fisher Scientific), and the cDNA was stored at - 20°C until use.

Quantitative real-time PCR (qPCR) was performed in 96-well PCR plates using a 7500Fast Real-Time PCR System (Applied Biosystems, Foster City, CA). The cDNA samples were diluted 10-fold and gene-specific transcription levels were determined in a 20 μl reaction volume in triplicates using TaqMan®Fast PCR Universal PCR Master mix following the manufacturer’s protocol. Commercially available primers and probe assays were from Applied Biosystems: *IL1B* (PN: Hs00174097_m1), *IL8* (PN: Hs00174103_m1), *FOS* (PN: Hs00170630_m1), *MYC* (PN: Hs00153408_m1), *TP53* (PN: Hs00153340_m1), and *CDKN1A* (PN: Hs00355782_m1), and *18S rRNA* (PN: Hs99999901_s1). The PCR cycling program included an enzyme activation step at 95°C for 20 seconds, and then 40 cycles of annealing and extension steps at 95°C for 3 seconds and 60°C for 30 seconds, respectively.

### Data analysis

The quantification cycle (*Cq*; standard name for Ct and Cp [16]) values were recorded with SDS v1.3 software (Applied Biosystems, Foster City, CA); the *Cq* value is the fractional cycle number at which the fluorescence exceeds a fixed threshold. The raw *Cq*-values were then exported into Excel- files and analyzed by the comparative *Cq* – method [17,18] using *18S rRNA* as internal control. Targets with *Cq*-values > 35 were considered beyond the limit of detection (LOD) and all *Cq*-values above 35 were coded as missing values. For downstream analysis missing values were replaced by *Cq* (LOD) + 1 (i.e., 35 + 1: *Cq* = 36). Target genes were normalized to *18S rRNA* internal controls, [this is given by Δ*Cq*; where Δ*Cq* (sample) = *Cq* (target gene) – *Cq* (internal controls)]. The ΔΔ*Cq* values were generated by subtracting the Δ*Cq*-value for the reference samples (calibrators) from the Δ*Cq*-value for the samples [ΔΔ*Cq* = Δ*Cq* (sample) – Δ*Cq* (calibrator); fold change = 2^-ΔΔ*Cq*^. A reference sample was determined within each study, and for samples without an obvious reference sample (calibrator), the linear scale of Δ*Cq* (sample); 2^-Δ*Cq*^ was used [17,18]. The fold change values were then log2-transformed in order to make the values symmetrical around zero. The *18S rRNA* stability was evaluated and results are presented in Additional file 4. These four genes: *CDKN1A*, *IL8*, *MYC*, and *TP53* were analyzed when the two RNA stabilization systems (PAXgene and Tempus) were compared, and then two more genes (*FOS* and *ILB*) were added, i.e. six genes were analyzed in the remaining study.

### Statistical analysis

Statistical analysis of RNA yield, purity, integrity and Δ*Cq* - values was carried out by one-way analysis of variance (ANOVA), followed by *post hoc* Dunnett’s tests to allow for multiple comparisons or by non-parametric Kruskal-Wallis test. Normal distribution and equality of variances was evaluated using the Shapiro-Wilk test and the Levene's test of homogeneity of variance. Two-sample t-test was used to compare cord blood samples collected in the PAXgene vs the Tempus tubes. The data are presented as means ± SE. Statistical analyses were performed using SPSS v17 software (SPSS, Inc., Chicago, IL), and p < 0.05 was accepted as statistically significant.

## Abbreviations

Cq: Quantification cycle Threshold; RT-qPCR: Quantitative Real-time PCR; RIN: RNA Integrity Number; MoBa: The Norwegian Mother and Child Cohort Study; NIPH: Norwegian Institute of Public Health; QC: Quality Control.

## Competing interests

The authors declare that they have no competing interests.

## Authors' contributions

ND participated in the study design and experimental work, participated in scientific discussions, carried out the data and the statistical analyses, interpreted the results, drafted the manuscript and prepared the finale version of the manuscript. GB participated in the study design, in scientific discussions, interpretation of the results, manuscript preparation and supervised the work. KR participated in the study design, in scientific discussions and in the experimental work. TB participated in the study design, in scientific discussions, and manuscript preparation. JA participated in the experimental work. KA participated in the experimental work. PM participated in scientific discussions and manuscript preparation. CS participated in scientific discussions and manuscript preparation. ES participated in scientific discussions and WL participated in the study design, in scientific discussions and manuscript preparation and co-supervised the work. All authors have read and approved the final version of the manuscript.

## Supplementary Material

Additional file 1 **The non-normalized raw *Cq*-values.** A) The non-normalized raw *Cq*-values for adult blood samples collected in the PAXgene tubes; B) The non-normalized raw *Cq*-values for adult blood samples collected in the Tempus tubes; C) The non-normalized raw *Cq*-values for cord blood samples collected in the PAXgene and in the Tempus tubes. Each bar represents the average *Cq*-values and the error bar indicates ± SE. Click here for file

Additional file 2 **Temperature recordings for transported samples vs RIN, RNA yield and Raw *Cq*-value.** Temperatures were monitored during the transportation of the samples to four different locations (hospitals) in Norway. The four samples were sent by standard mail at February 21^th^ and all samples returned to NIPH at February 24^th^. Transportation time for samples sent to Bærum, Kristiansand and Bergen was ~ 48 hours, while it took ~ 74 hours for Kirkenes samples to return to NIPH. The temperature variations ranged from 3.4°C to 27.1°C, and none of the samples were frozen during the transportation. A) Temperature recordings for samples sent to the four locations and samples kept at NIPH; B) RIN values vs. average temperature (°C) of the samples; C) RNA yields vs. average temperature (°C) of the samples; D) The non-normalized raw Cq-values vs. average temperature (°C) of the samples. Each bar represents the average Cq-values and the error bar indicates ± SE for the raw Cq-values or ± SD for average temperature (°C) of the samples. Click here for file

Additional file 3 **The non-normalized raw *Cq*-values for suboptimal blood volume QC.** A) Evaluation of the non-normalized raw *Cq*-values for suboptimal blood volume QC from adult blood samples; and B) Evaluation of the non-normalized raw *Cq*-values for suboptimal blood volume QC from cord blood samples. Each bar represents the average *Cq*-values and the error bar indicates ± SE. Click here for file

Additional file 4 **The evaluation of *18S rRNA *stability.** A) Comparison of PAXgene tube-collected samples against the Tempus tube-collected samples: The *Cq* – values for *18S rRNA* were relatively stable following the storage of the tubes for up to seven days at room temperature for both tubes; B) Evaluation of 18S *rRNA* stability for transportation QC samples: The *Cq* – values for *18S rRNA* were relatively stable following the transportation; C) Suboptimal blood volume in the Tempus tubes on *18S rRNA* expression stability for adult blood samples: Suboptimal blood volume collected in the tube has a significant effects on *18S rRNA* expression stability; and D) Suboptimal blood volume in the Tempus tubes on *18S rRNA* expression stability for cord blood samples: Suboptimal blood volume collected in the tube has some effects on *18S rRNA* expression stability. Each bar represents the average *Cq*-values and the error bar indicates ± SE. Click here for file
