# Supplementary material for: Human blood RNA stabilization in samples collected and transported for a large biobank
Source: BMC Res Notes. 2012 Sep 18;5:510. doi: 10.1186/1756-0500-5-510 (PMC3503553; doi:10.1186/1756-0500-5-510)

Additional file 2 – The non-normalized raw Cq-values for PAXgene vs Tempus tubes

A) The non-normalized raw Cq-values for adult blood samples collected in the PAXgene tubes; B) The non-normalized raw Cq-values for adult blood samples collected in the Tempus tubes; C) The non-normalized raw Cq-values for cord blood samples collected in the PAXgene and in the Tempus tubes. Each bar represents the average *Cq*-values and the error bar indicates ± SE.

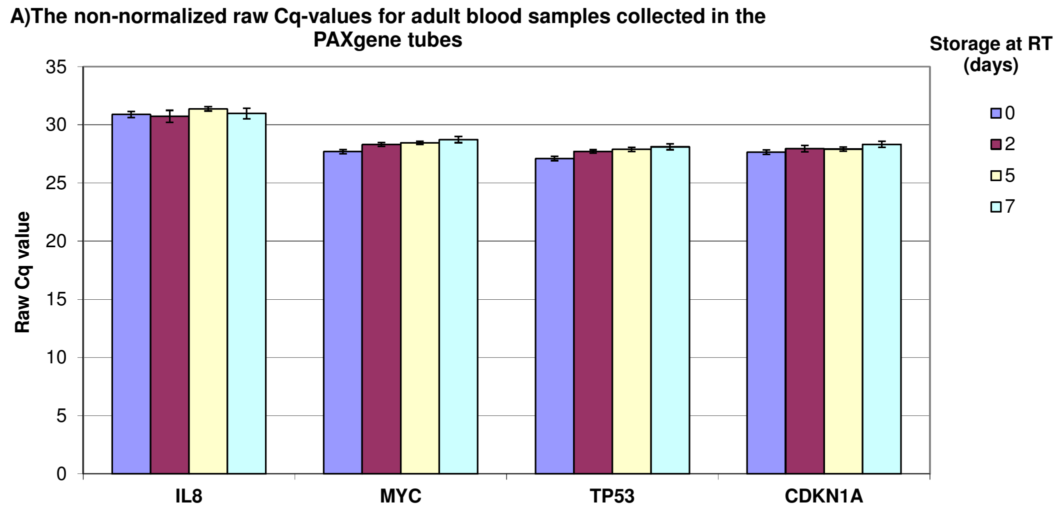

	IL8	MYC	TP53	CDKN1A
Average ± SD	31.0 ± 0.45	28.3 ± 0.41	27.7 ± 0.41	28.0 ± 0.33
(min - max)	(30.1 - 31.7)	(27.4 - 29.3)	(26.7 - 28.6)	(27.4 - 29.1)

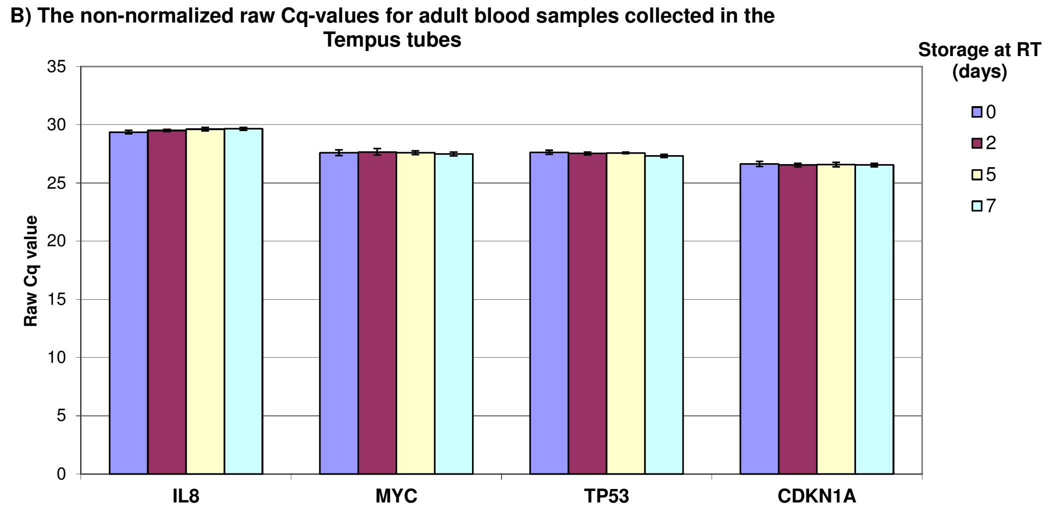

	IL8	MYC	TP53	CDKN1A
Average ± SD	29.5 ± 0.17	27.6 ± 0.22	27.5 ± 0.18	26.6 ± 0.18
(min - max)	(29.1 - 29.8)	(27.2 - 28.4)	(27.1 - 27.9)	(26.2 - 27.1)

C) The non-normalized raw Cq-values for cord blood samples collected in the PAXgene and in the Tempus tubes

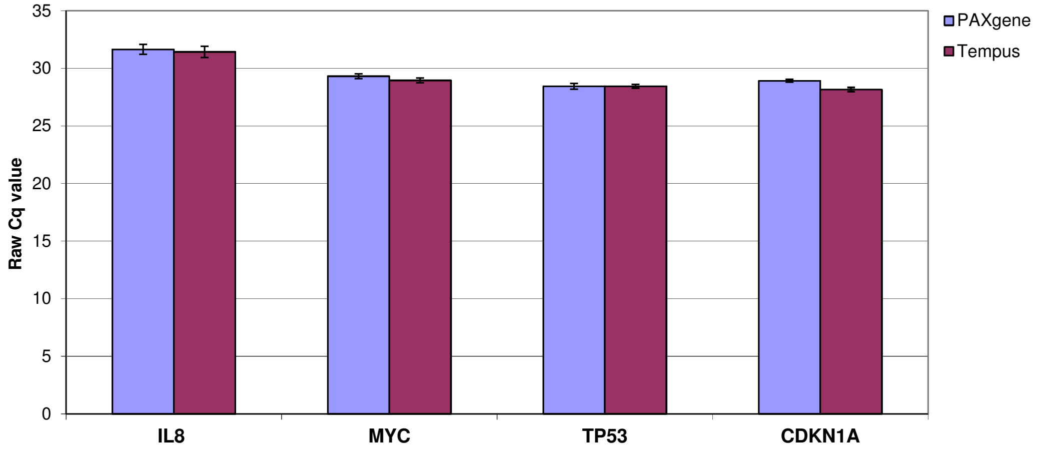

Supplement: Additional file 1 — The non-normalized raw Cq-values. A) The non-normalized raw Cq-values for adult blood samples collected in the PAXgene tubes; B) The non-normalized raw Cq-values for adult blood samples collected in the Tempus tubes; C) The non-normalized raw Cq-values for cord blood samples collected in the PAXgene and in the Tempus tubes. Each bar represents the average Cq-values and the error bar indicates ± SE. [file 1756-0500-5-510-S1.pdf]
