# Supplementary material for: Human blood RNA stabilization in samples collected and transported for a large biobank
Source: BMC Res Notes. 2012 Sep 18;5:510. doi: 10.1186/1756-0500-5-510 (PMC3503553; doi:10.1186/1756-0500-5-510)
Supplement: Additional file 2 — Temperature recordings for transported samples vs RIN, RNA yield and Raw Cq-value. Temperatures were monitored during the transportation of the samples to four different locations (hospitals) in Norway. The four samples were sent by standard mail at February 21th and all samples returned to NIPH at February 24th. Transportation time for samples sent to Bærum, Kristiansand and Bergen was ~ 48 hours, while it took ~ 74 hours for Kirkenes samples to return to NIPH. The temperature variations ranged from 3.4°C to 27.1°C, and none of the samples were frozen during the transportation. A) Temperature recordings for samples sent to the four locations and samples kept at NIPH; B) RIN values vs. average temperature (°C) of the samples; C) RNA yields vs. average temperature (°C) of the samples; D) The non-normalized raw Cq-values vs. average temperature (°C) of the samples. Each bar represents the average Cq-values and the error bar indicates ± SE for the raw Cq-values or ± SD for average temperature (°C) of the samples. [file 1756-0500-5-510-S2.pdf]

## Additional file 3 – Temperature recordings for transported samples vs RIN, RNA yield and Raw *Cq*-value

Temperatures were monitored during the transportation of the samples to four different locations (hospitals) in Norway. The four samples were sent by standard mail at February 21th and all samples returned to NIPH at February 24th. Transportation time for samples sent to Bærum, Kristiansand and Bergen was ~ 48 hours, while it took ~ 74 hours for Kirkenes samples to return to NIPH. The temperature variations ranged from 3.4°C to 27.1 °C, and none of the samples were frozen during the transportation. A) Temperature recordings for samples sent to the four locations and samples kept at NIPH; B) RIN values vs. average temperature (°C) of the samples; C) RNA yields vs. average temperature (°C) of the samples; D) The non-normalized raw Cq-values vs. average temperature (°C) of the samples. Each bar represents the average Cq-values and the error bar indicates ± SE for the raw Cq-values or ± SD for average temperature (°C) of the samples.

**A) Temperature log for transported samples**

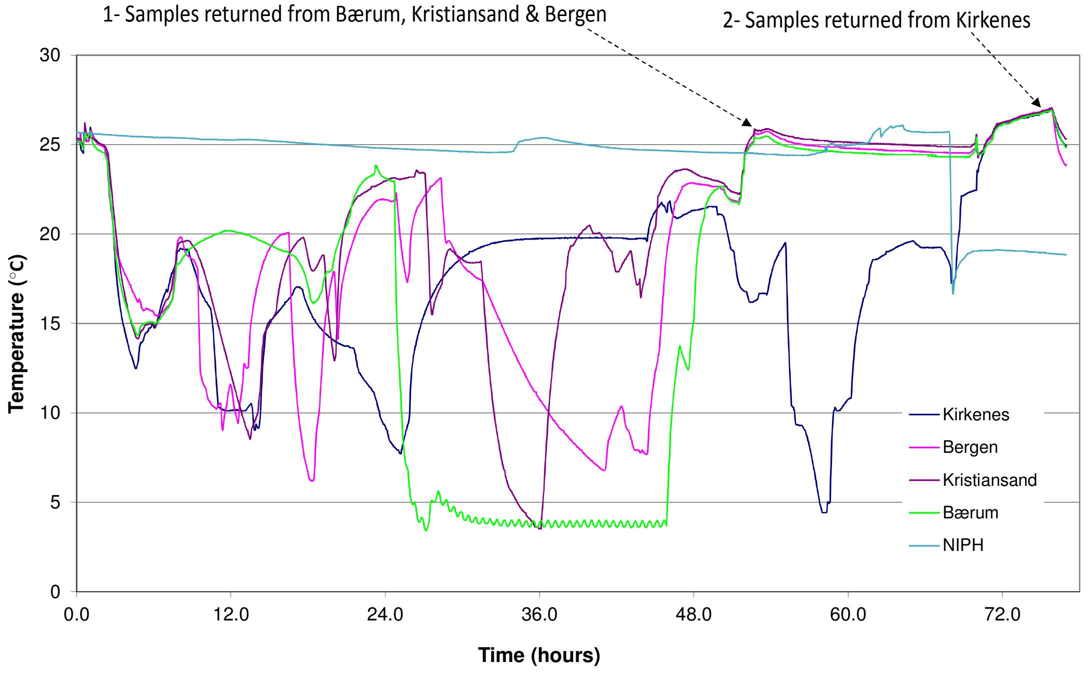


| | Kirkenes | Bergen | Kristiansand | Bærum | NIPH |
|---|---|---|---|---|---|
| Mean temp. (ºC) ± SD | 17.6 ± 4.9 | 19.1 ± 6.1 | 20.3 ± 5.6 | 17.0 ± 8.5 | 24.4 ± 1.9 |
| (Min - Max) | (4.4 - 27.0) | (6.2 - 27.1) | (3.5 - 27.0) | (3.4 - 26.9) | (16.6 - 26.1) |
| Average temp. difference between NIPH & transported samples | 7.4 | 5.9 | 4.7 | 8.0 | 0 |

| Averaged temperature (ºC) recordings divided into 12-hour intervals | | | | | | |
|---|---|---|---|---|---|---|
| | **0h - 12h** | **12h - 24h** | **24h - 36h** | **36h - 48h** | **48h - 60h** | **60h - 76h** |
| **Kirkenes** | 17.6 | 13.3 | 16.7 | 20.2 | 14.8 | 21.5 |
| **Bergen** | 17.7 | 16.0 | 17.8 | 11.5 | 24.3 | 25.2 |
| **Kristiansand** | 18.6 | 17.2 | 15.4 | 18.8 | 24.6 | 25.4 |
| **Bærum** | 19.2 | 19.8 | ***6.0*** | ***5.2*** | 23.8 | 25.1 |
| **NIPH** | 25.4 | 25.1 | 24.7 | 24.9 | 24.6 | 22.2 |

**B) RIN-value vs average temperature recordings**

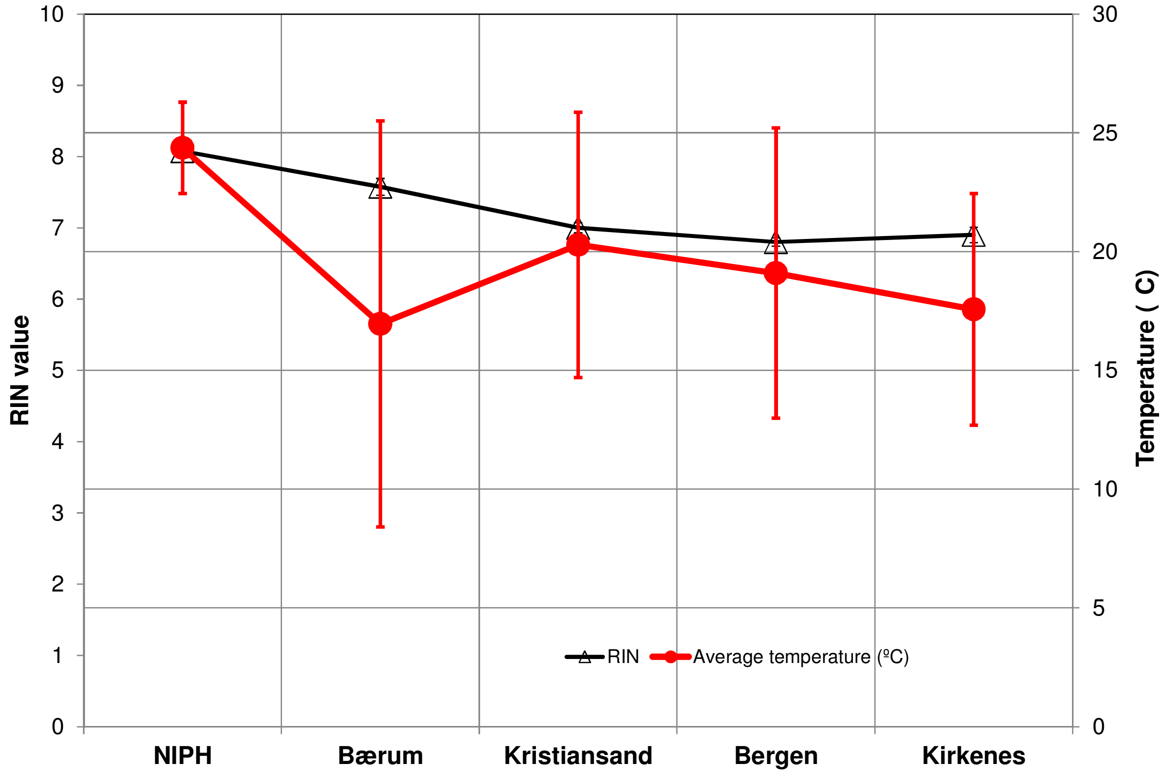

C) RNA yield vs average temperature recordings

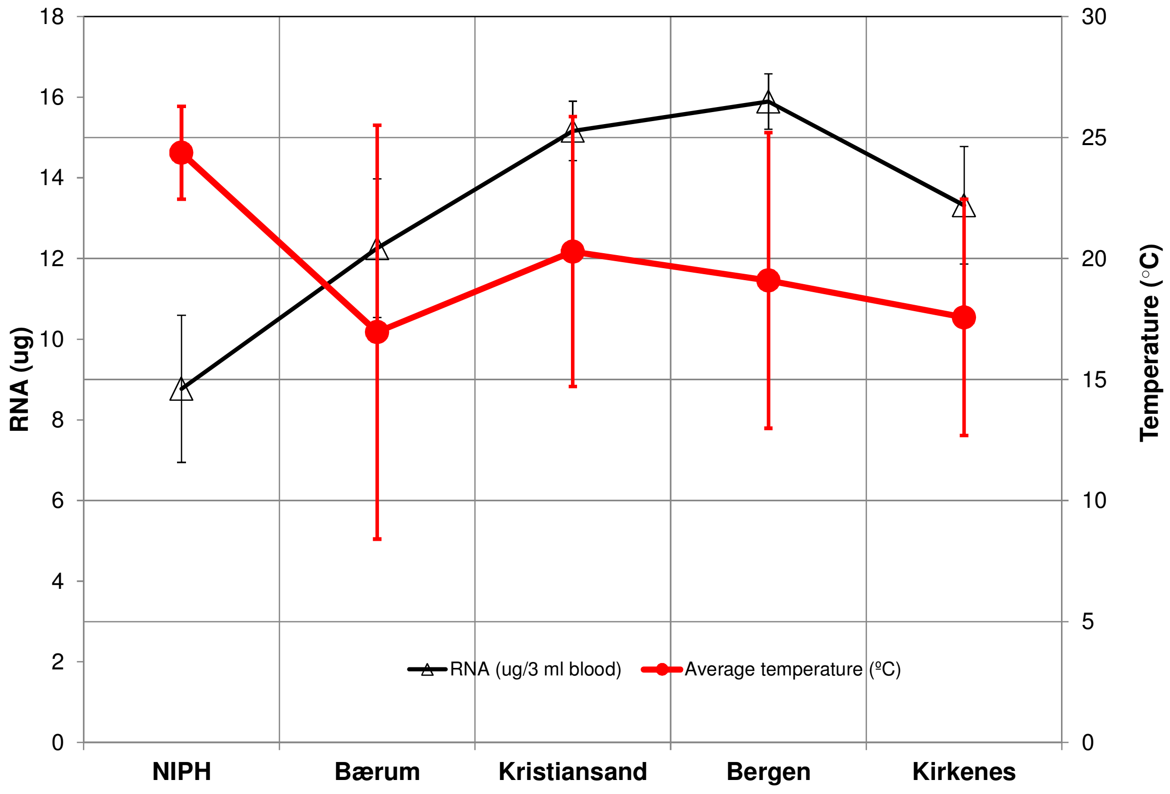

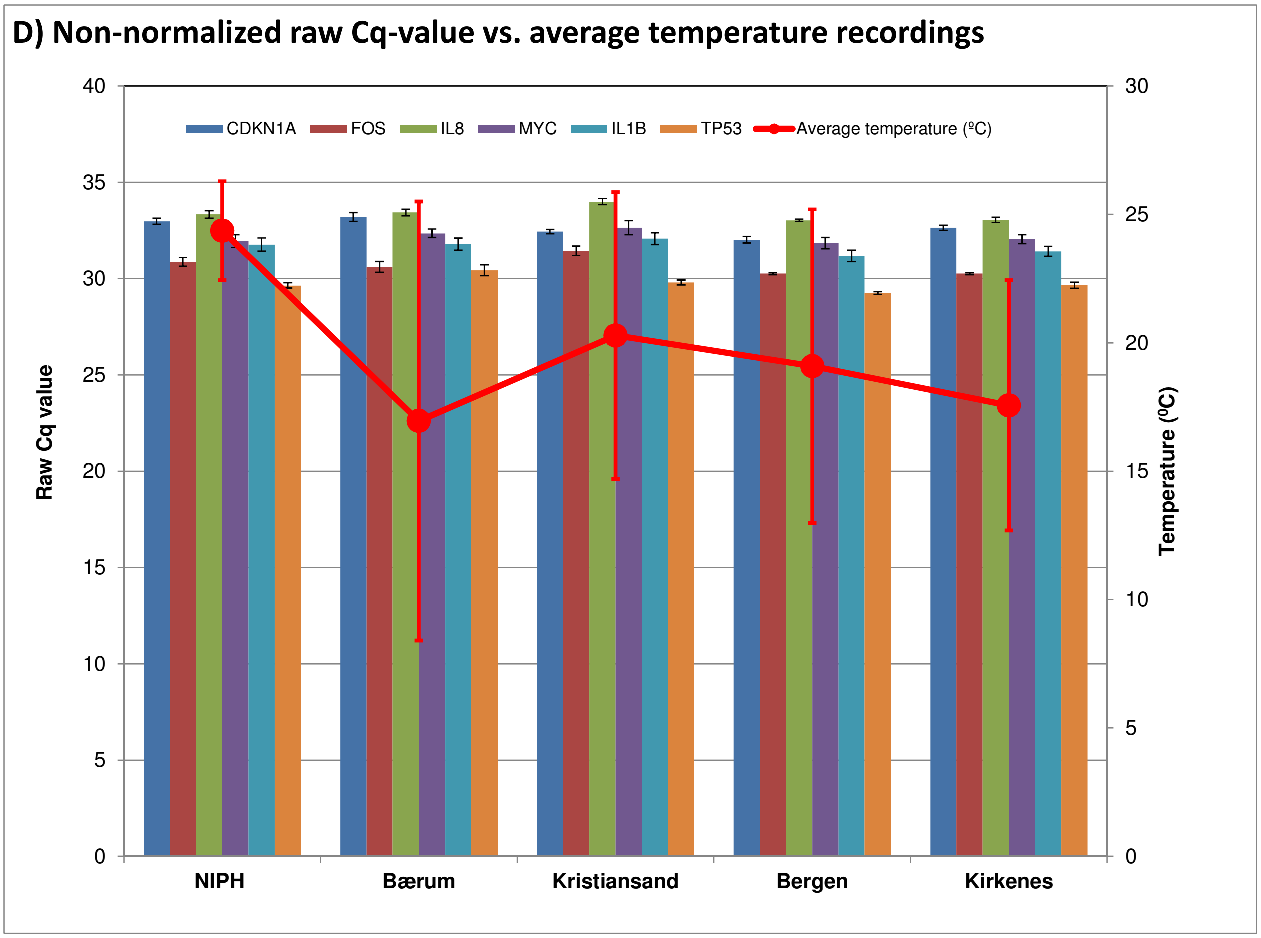
D) Non-normalized raw Cq-value vs. average temperature recordings
CDKN1A
FOS
IL8
MYC
IL1B
TP53
Average temperature (ºC)
Raw Cq value
Temperature (ºC)
0
5
10
15
20
25
30
35
40
0
5
10
15
20
25
30
NIPH
Bærum
Kristiansand
Bergen
Kirkenes
