# Supplementary material for: Human blood RNA stabilization in samples collected and transported for a large biobank
Source: BMC Res Notes. 2012 Sep 18;5:510. doi: 10.1186/1756-0500-5-510 (PMC3503553; doi:10.1186/1756-0500-5-510)
Supplement: Additional file 3 — The non-normalized raw Cq-values for suboptimal blood volume QC. A) Evaluation of the non-normalized raw Cq-values for suboptimal blood volume QC from adult blood samples; and B) Evaluation of the non-normalized raw Cq-values for suboptimal blood volume QC from cord blood samples. Each bar represents the average Cq-values and the error bar indicates ± SE. [file 1756-0500-5-510-S3.pdf]

Additional file 4 – The non-normalized raw *Cq*-values for suboptimal blood volume QC

A) Evaluation of the non-normalized raw *Cq*-values for suboptimal blood volume QC from adult blood samples; and B) Evaluation of the non-normalized raw *Cq*-values for suboptimal blood volume QC from cord blood samples. Each bar represents the average *Cq*-values and the error bar indicates ± SE.

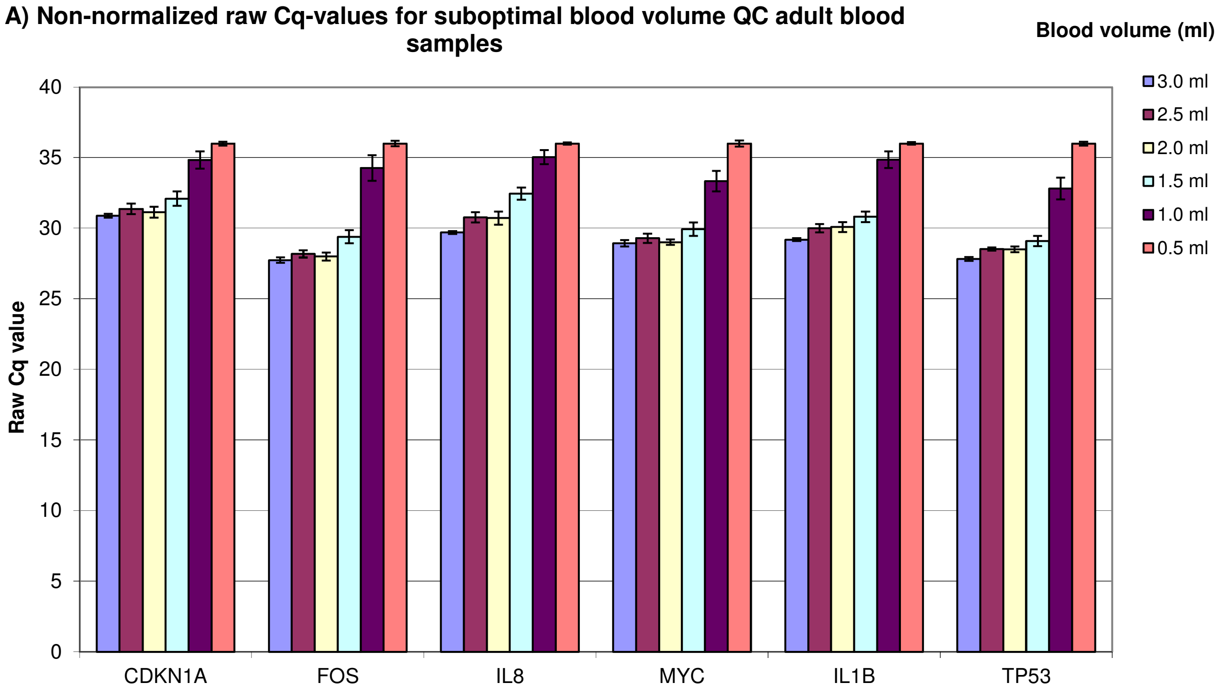

	CDKN1A	FOS	IL8	MYC	IL1B	TP53
Average ± SD	32.7 ± 2.31	30.5 ± 3.56	32.4 ± 2.57	30.9 ± 2.83	31.8 ± 2.81	30.3 ± 3.04
(mn - max)	(29.0 - 36.0)	(26.9 - 36.0)	(28.6 - 36.0)	(26.9 - 36.0)	(28.4 - 36.0)	(27.3 - 36.0)

B) Non-normalized raw Cq-values for suboptimal blood volume QC cord blood samples

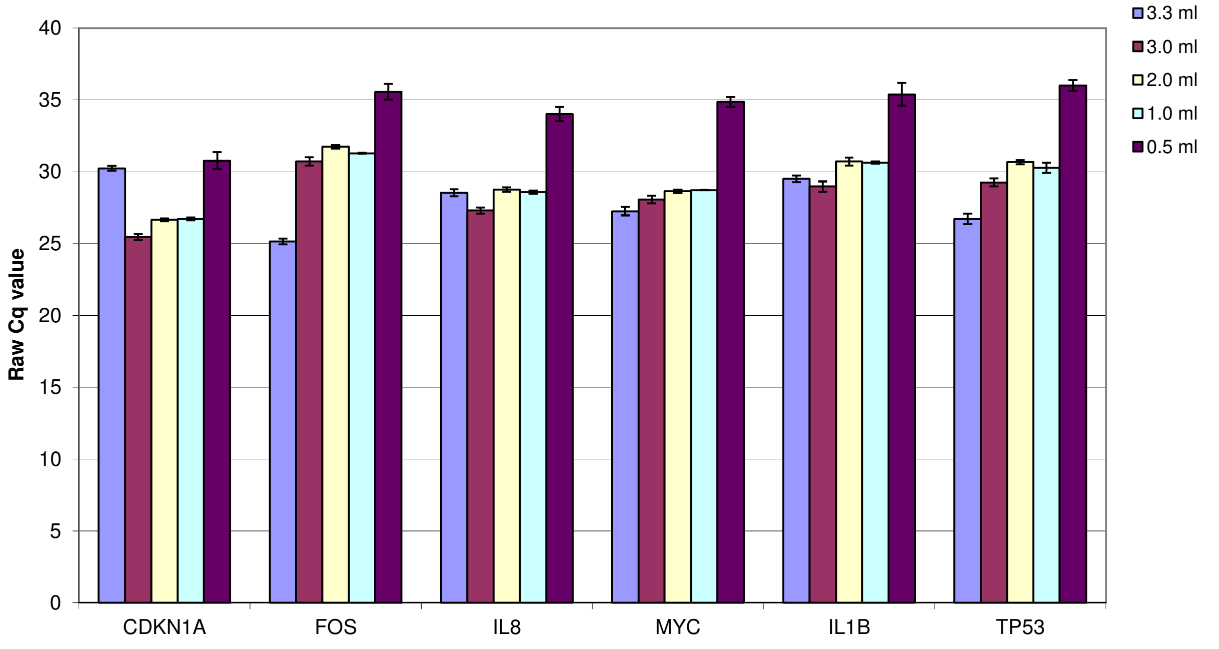

	CDKN1A	FOS	IL8	MYC	IL1B	TP53
Average ± SD	27.7 ± 2.10	30.4 ± 3.20	28.9 ± 1.97	28.9 ± 2.31	30.6 ± 2.00	30.0 ± 2.73
(min - max)	(24.5 - 31.6)	(24.5 - 36.0)	(26.2 - 34.3)	(26.6 - 36.0)	(28.2 - 36.0)	(26.1 - 36.0)
