# Supplementary material for: Human blood RNA stabilization in samples collected and transported for a large biobank
Source: BMC Res Notes. 2012 Sep 18;5:510. doi: 10.1186/1756-0500-5-510 (PMC3503553; doi:10.1186/1756-0500-5-510)
Supplement: Additional file 4 — The evaluation of 18S rRNA stability. A) Comparison of PAXgene tube-collected samples against the Tempus tube-collected samples: The Cq – values for 18S rRNA were relatively stable following the storage of the tubes for up to seven days at room temperature for both tubes; B) Evaluation of 18S rRNA stability for transportation QC samples: The Cq – values for 18S rRNA were relatively stable following the transportation; C) Suboptimal blood volume in the Tempus tubes on 18S rRNA expression stability for adult blood samples: Suboptimal blood volume collected in the tube has a significant effects on 18S rRNA expression stability; and D) Suboptimal blood volume in the Tempus tubes on 18S rRNA expression stability for cord blood samples: Suboptimal blood volume collected in the tube has some effects on 18S rRNA expression stability. Each bar represents the average Cq-values and the error bar indicates ± SE. [file 1756-0500-5-510-S4.pdf]

Additional file 1- The evaluation of 18S rRNA expression stability

A) Comparison of PAXgene tube-collected samples against the Tempus tube-collected samples: The Cq – values for 18S rRNA were relatively stable following the storage of the tubes for up to seven days at room temperature for both tubes; B) Evaluation of 18S rRNA stability for transportation QC samples: The Cq – values for 18S rRNA were relatively stable following the transportation; C) Suboptimal blood volume in the Tempus tubes on 18S rRNA expression stability for adult blood samples: Suboptimal blood volume collected in the tube has a significant effects on 18S rRNA expression stability; and D) Suboptimal blood volume in the Tempus tubes on 18S rRNA expression stability for cord blood samples: Suboptimal blood volume collected in the tube has some effects on 18S rRNA expression stability. Each bar represents the average Cq-values and the error bar indicates ± SE.

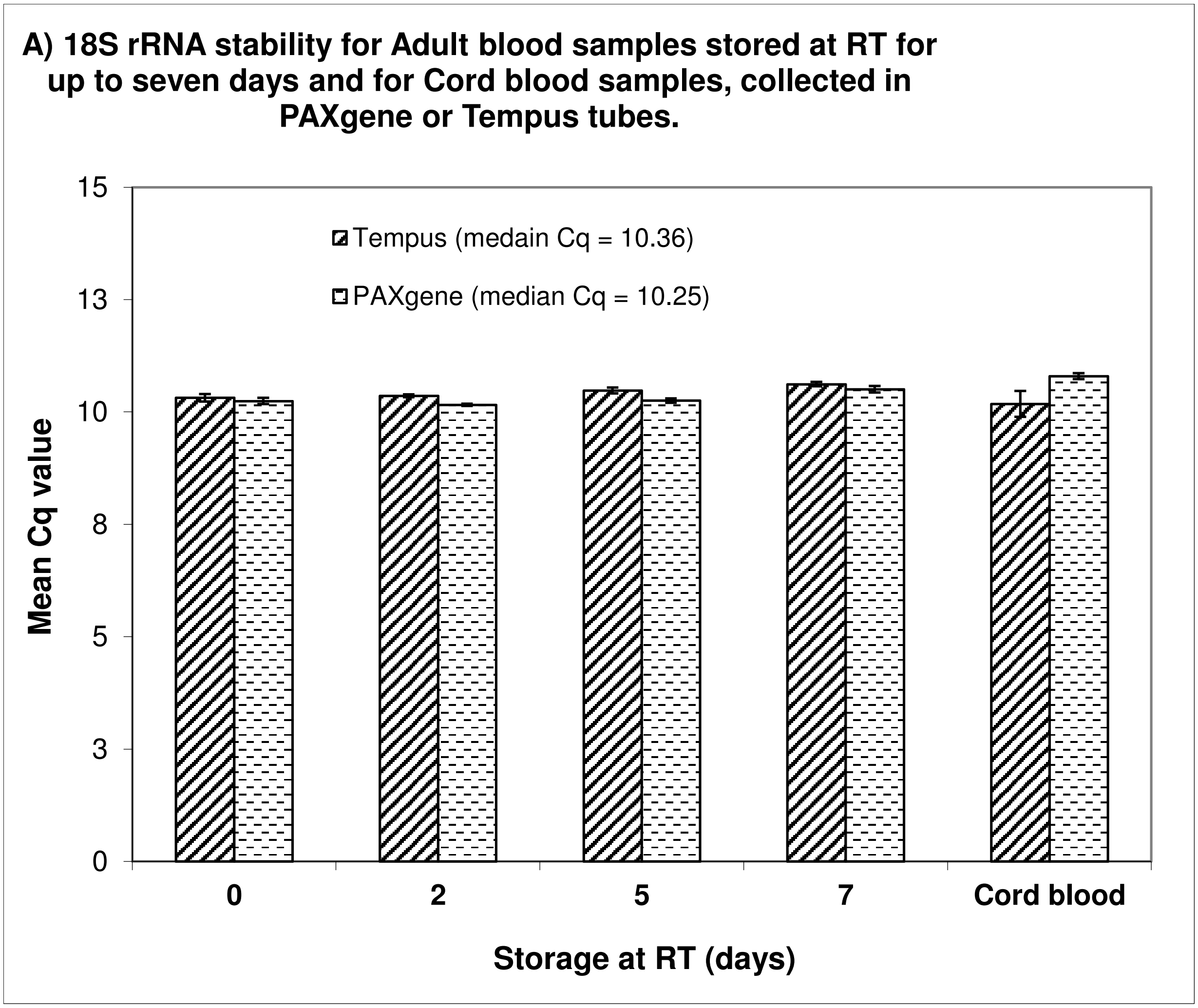

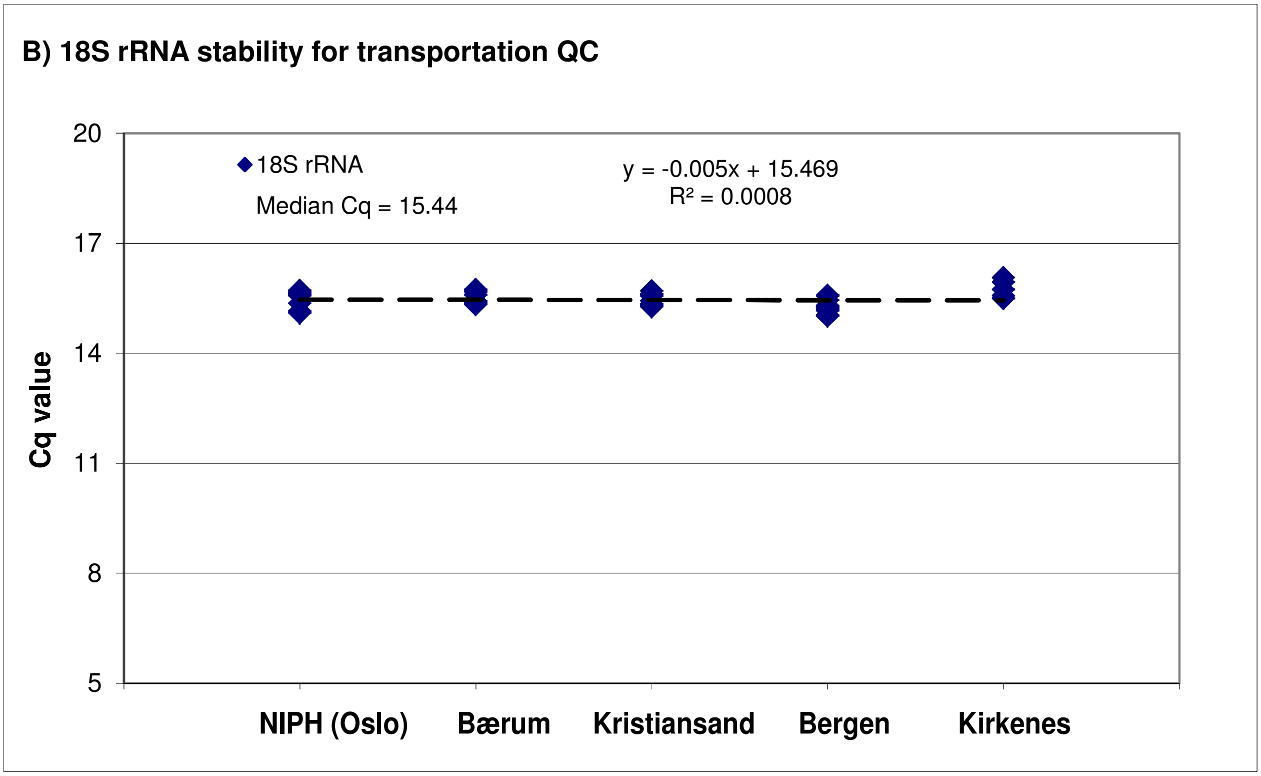
B) 18S rRNA stability for transportation QC
18S rRNA
Median Cq = 15.44
y = -0.005x + 15.469
R² = 0.0008
Cq value
20
17
14
11
8
5
NIPH (Oslo)
Bærum
Kristiansand
Bergen
Kirkenes

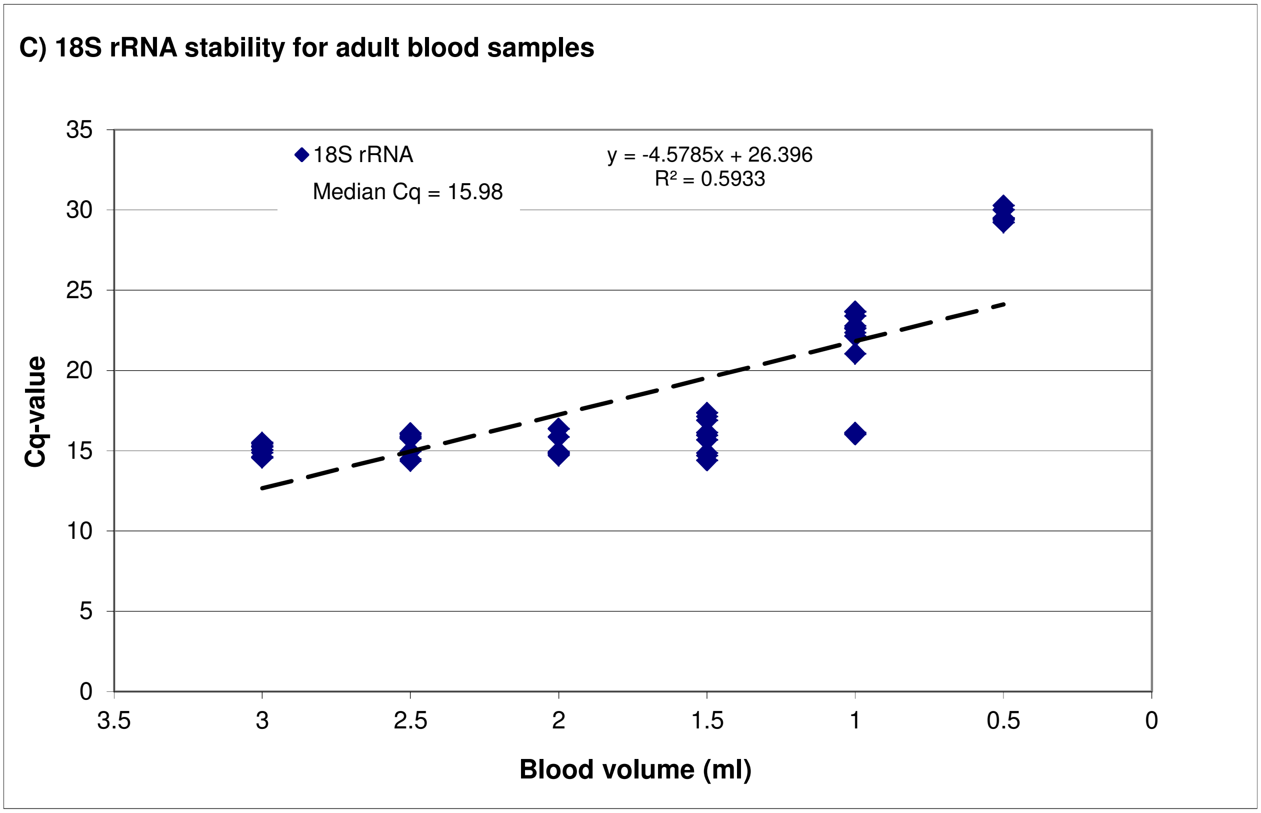
C) 18S rRNA stability for adult blood samples
18S rRNA
Median Cq = 15.98
y = -4.5785x + 26.396
R² = 0.5933
Cq-value
35
30
25
20
15
10
5
0
3.5
3
2.5
2
1.5
1
0.5
0
Blood volume (ml)

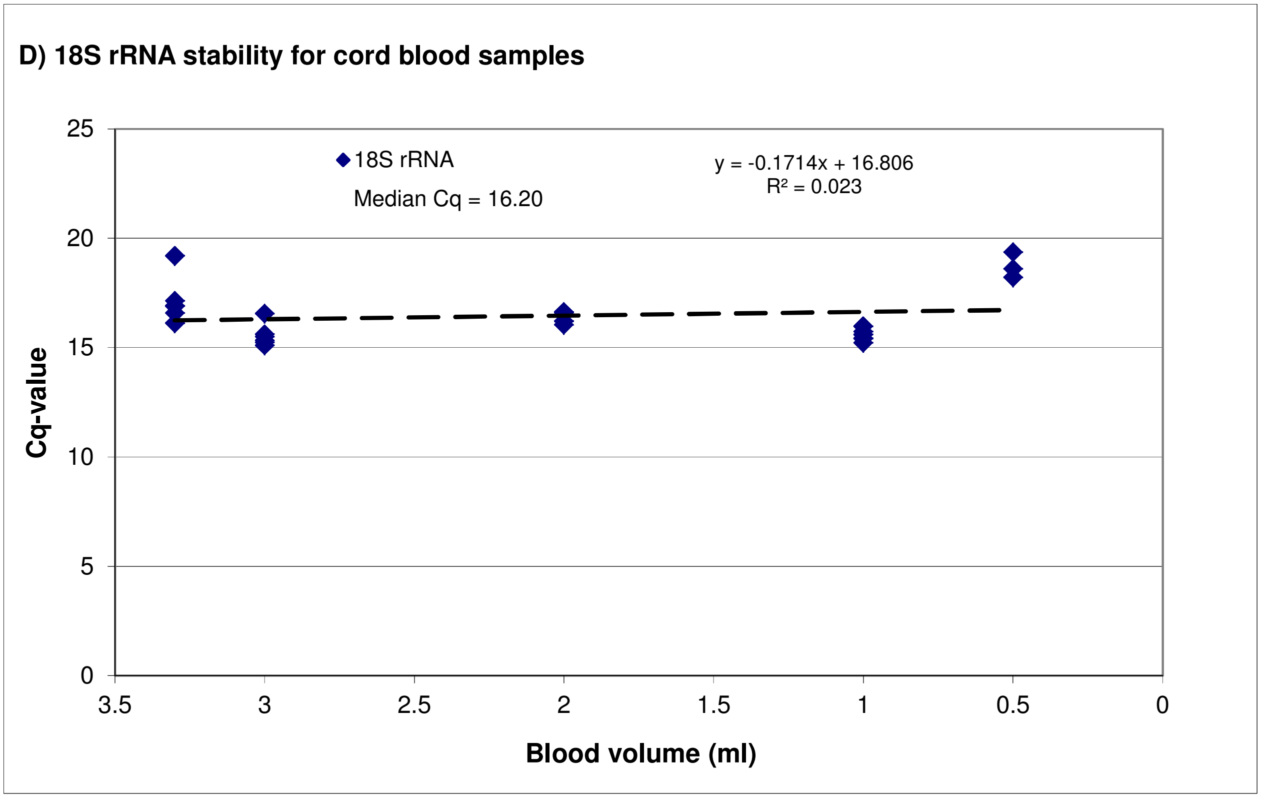
D) 18S rRNA stability for cord blood samples
18S rRNA
Median Cq = 16.20
y = -0.1714x + 16.806
R² = 0.023
Cq-value
25
20
15
10
5
0
3.5
3
2.5
2
1.5
1
0.5
0
Blood volume (ml)
